# Anesthetic Management and Neuromonitoring in a Patient with Very Long-Chain Acyl-Coenzyme A Dehydrogenase Deficiency Undergoing Scoliosis Surgery: A Case Report and Review of Literature

**DOI:** 10.1155/2024/1050279

**Published:** 2024-01-08

**Authors:** Anna Tanaka, Tim Cai, Michael Platten, Luis E. Tollinche, Samuel J. DeJoy

**Affiliations:** ^1^Case Western Reserve University School of Medicine, Cleveland, OH, USA; ^2^Department of Anesthesiology, MetroHealth Medical Center of Case Western Reserve University School of Medicine, Cleveland, OH, USA

## Abstract

Patients with very long-chain acyl-CoA dehydrogenase deficiency (VLCADD) are prone to hypoglycemia and clinical decompensation when metabolic demands of the body are not met. We present a pediatric patient with VLCADD who underwent a posterior spinal fusion for scoliosis requiring intraoperative neurophysiology monitoring. Challenges included minimization of perioperative metabolic stressors and careful selection of anesthetic agents since propofol-based total intravenous anesthesia (TIVA) was contraindicated due to its high fatty acid content. This case is unique due to the sequential use of inhaled anesthetics after TIVA to allow for a rapid wakeup and immediate postoperative physical exam. Additionally, intraoperative neuromonitoring in the setting of VLCADD has not been reported in the literature. With communication among anesthesia, surgery, and neuromonitoring teams before and during the operation, the patient successfully underwent a major surgery without complications. This trial is registered with NCT03808077.

## 1. Introduction

Very long-chain acyl-coenzyme A dehydrogenase (VLCAD) deficiency, or VLCADD, is an autosomal recessive disorder of impaired fatty acid metabolism. VLCAD catalyzes the breakdown of 14 to 20 carbon long-chain fatty acids during beta-oxidation [1]. In times of increased metabolic demand or fasting, VLCADD patients face deficient energy supply and accumulation of toxic fatty acid metabolites that can damage multiple organ systems [2]. Stress, cold exposure, pain, and illness are mechanisms by which the body requires an increased energy supply and are commonly encountered by a patient perioperatively. We describe the anesthetic management of a 13-year-old patient with VLCADD who underwent a posterior spinal fusion for scoliosis. Due to the need for intraoperative motor and somatosensory neuromonitoring and the patient's contraindication to propofol, unique anesthetic consideration was needed; total intravenous anesthesia (TIVA) and inhaled anesthetics were used sequentially for the management of this patient. This manuscript adheres to the CARE guidelines and is patient privacy compliant per our institution's guidelines with written Health Insurance Portability and Accountability Act (HIPPA) authorization form completed.

### 1.1. Case Description

A 13-year-old female (weight 56 kg, BMI 22) with past medical history of VLCADD, mild intermittent asthma, no past surgical history, and ASA physical status class III underwent a posterior spinal fusion and segmental spinal instrumentation of T3-L3, along with Ponte osteotomies at T5-L2 for severe adolescent idiopathic scoliosis. As a newborn, she had recurrent episodes of hypoglycemia requiring prolonged neonatal ICU admission. Her diagnosis was made with a positive newborn screen followed by VLCAD gene sequencing showing 2 deleterious mutations (C1748G (S583W) and C1894T (R632C)). Preoperatively, cardiovascular review of systems was negative. Creatine kinase (CK) and renal function tests were within normal limits.

During surgery, in addition to standard American Society of Anesthesiologists' (ASA) monitoring, invasive blood pressure monitoring was achieved with radial artery cannulation. A raw 4-channel EEG was used for neuromonitoring. The patient was premedicated with 4 mg of midazolam and induction was achieved using 20 mcg/kg fentanyl, 3 mg/kg ketamine, 1.5 mg/kg lidocaine, and 0.5 mg/kg rocuronium before intubation. A high dose of fentanyl was used to provide a strong analgesic to prevent movement intraoperatively; a neuromuscular blocking agent could not be used due to the need for motor-evoked potential (MEP) monitoring. Although ketamine is also an analgesic, it does not last long enough in a single dose at induction to provide the high level of analgesia needed for the duration of this surgery. Infusions of ketamine at 1.0 mg/kg/hr, lidocaine at 1.5 mg/kg/hr, midazolam at 3.0 mg/hr, and remifentanil at 0.15 mcg/kg/hr were administered for maintenance of anesthesia and analgesia. The rocuronium did not need reversal as the patient had 3 out of 4 MEPs before the procedure began. Boluses of anesthetics were avoided to preserve the neuromonitoring signals at a steady state, since the latency and amplitude of signals risk being increased and decreased, respectively.

Per our enhanced recovery after surgery (ERAS) protocol, the patient ingested a clear carbohydrate-rich supplement 3 hours before the procedure (Nestle BOOST Breeze®, 237 mL, 54 g total carbohydrates). Intraoperatively, a background infusion of dextrose 5% in lactated Ringer's solution was run at 110 mL/hr to prevent hypoglycemia, while glucose and CK levels were monitored closely to detect potential rhabdomyolysis early (Table 1). The patient's temperature was maintained within a range of 36–38C° using warmed fluid, increased ambient temperature, and forced air warmers set to 43C° (±3C°).

Because neuromonitoring is not definitive for determining neurological damage, a physical exam is necessary at the end of the surgery. After successful derotation of the spine and confirmation of somatosensory evoked potential (SSEP) and MEP signals, the surgeons began closing the incision. At that time, the infusions of ketamine and midazolam were stopped, and inhaled anesthetics were started for a more predictable wakeup in preparation for the planned physical exam after wakeup and extubation.

**Figure 1:**
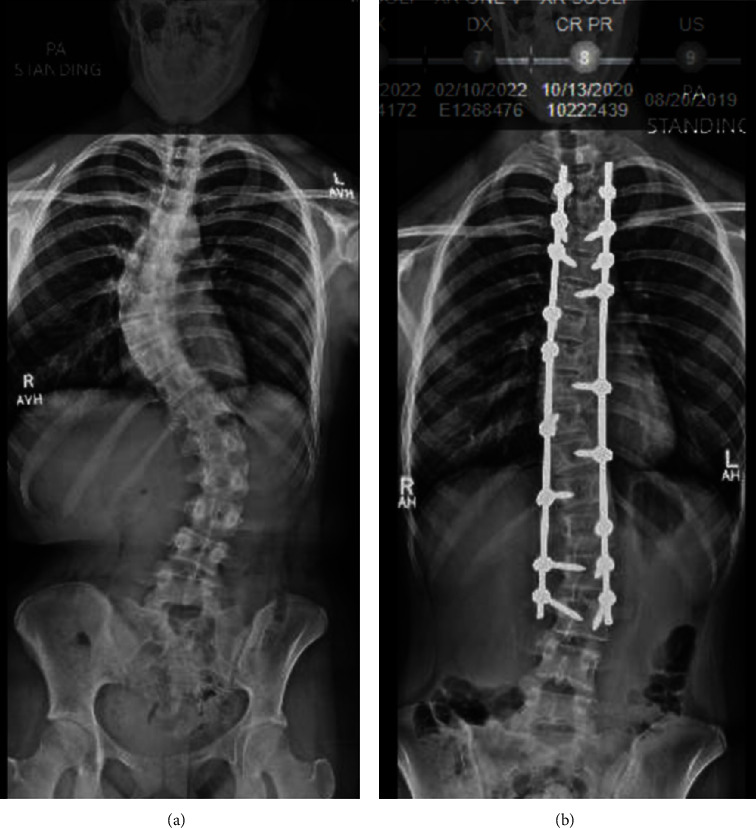
Patient's successful segmental spinal instrumentation seen on X-ray. (a) Before the procedure and (b) after the procedure.

Total surgery time was 3 h16 m and total anesthesia time was 4 h18 m. Estimated blood loss was 1200 mL and total fluids given were IV fluid 3.5 L crystalloid and 500 mL 5% albumin. Urine output was 600 mL. Intraoperative fluid management remains at the discretion of the attending anesthesiologist; however, goal directed management is strongly encouraged and, as in this case, generally adopted. Several variables inform fluid management including blood loss, urine output, blood pressure, insensible loss from the wound, and the patient's physiologic response to prone positioning. Due to decreased venous return from pooling in the viscera and lower extremities, prone positioning necessitates adequate preload to maintain blood pressure; this is most successful with judicious administration of balanced crystalloids and/or colloids. Replacement with 3 mLs of crystalloid per 1 mL of blood loss was targeted. Cell saver was used for the procedure and allogenic blood transfusion was not given. Postoperatively, the patient was able to move all extremities on physical exam. Dexmedetomidine was administered for immediate postanesthesia care unit (PACU) analgesia. The patient stayed in the PACU for 2 hours before being transferred to the postoperative floor, where dexmedetomidine was discontinued and multimodal analgesia of acetaminophen, gabapentin, ketorolac, and opioids were used. The patient was discharged on postoperative day 4. Figures 1(a) and 1(b) show x-ray imaging of the successful procedure.

## 2. Discussion

VLCADD is a rare disorder with an estimated incidence of 1 : 30,000–400,000 live births [3]. Due to deficient or defective VLCAD enzyme, these patients are unable to break down fatty acids to utilize as a secondary energy source when glucose levels are low, and thus are prone to hypoglycemia [4, 5]. The clinical severity of symptoms varies greatly among patients: some present with multisystemic disease and organ failure, while others only exhibit mild clinical symptoms with vigorous exercise or illness [2]. During periods of unmet energy demands, catabolic processes such as rhabdomyolysis, cardiac dysfunction, or arrhythmias can occur, manifesting clinically as symptoms such as muscle pain, cramps, and weakness [1, 2]. Additionally, when long-chain fatty acids remain unmetabolized, cytotoxic long-chain acyl-carnitines accumulate in various organ systems, which can cause cardiomyopathy, skeletal myopathy, and organ lipidosis [6, 7]. Because of these potentially life-threatening complications, patients with VLCADD require dietary modifications including scheduling regularly spaced meals, avoiding long-chain fatty acids, and supplementing with medium-chain fatty acids [8]. By maintaining a constant glucose supply, the need for beta-oxidation as a source of energy is prevented.

Given these characteristics, our main goal during this procedure was to avoid extraneous stressors that would increase metabolic demand and cause decompensation in the patient. This was done while simultaneously considering the need for adequate intraoperative neuromonitoring conditions needed for a safe scoliosis surgery. Importantly, motor-evoked potential monitoring is crucial for the early recognition of potential or impending neurological damage in these pediatric patients. Spinal cord ischemia or direct cord injury during the procedure can have devastating consequences for the patient. Although propofol is typically used as part of the TIVA technique during surgeries requiring neuromonitoring, it is contraindicated for VLCADD patients due to its emulsion consisting of mainly long-chain fatty acids that the patient would not be able to properly metabolize [1, 4]. Small induction dosages of propofol may be tolerated in some VLCADD patients depending on the degree of the enzyme deficiency or in the presence of sufficient glucose supply. Conversely, propofol must be avoided entirely given its potential for severe complications in VLCADD patients [4]. TIVA technique with propofol was excluded given the dose dependent increase in sequelae from propofol administration. An alternative anesthetic plan of fentanyl, ketamine, and lidocaine was used for induction, and ketamine, lidocaine, midazolam and remifentanil were infused intraoperatively for anesthetic maintenance. Narcotic target-controlled infusions must reach a threshold concentration to prevent intraoperative patient movement. In lieu of waiting for target concentration of remifentanil, and to ensure adequate analgesia, fentanyl boluses were administered at induction. To avoid elevations in CK that can be caused by depolarizing neuromuscular blocking agents, rocuronium, a nondepolarizing agent, was used to optimize our intubation before the start of the procedure [4]. Notably, low-dose dexmedetomidine was not utilized as part of the TIVA due to the drug's effect on suppressing MEPs. Mahmoud et al., as well as a more recent retrospective case-control study by Holt et al., showed clinically and statistically significant attenuation of MEP amplitudes using dexmedetomidine during TIVA for posterior spinal fusion surgeries [9, 10]. Dexmedetomidine was, however, used postoperatively because of its advantageous effects as an analgesic and sedative during the recovery period from a major surgery.

Our anesthetic management was unique since TIVA was used for the majority of the surgery, while inhaled anesthetics were subsequently used near the end of the procedure after stopping infusions. This method was advantageous in this situation given the need to carefully time the patient's awakening and consider the depth of the patient's anesthesia so an adequate physical exam could be performed after wakeup. Additionally, a potential intraoperative Stagnara test also necessitates a rapid wakeup. Due to the rapid metabolism of remifentanil by plasma esterases and the ability to reverse the effects of both fentanyl and midazolam intraoperatively via titration of intravenous naloxone and flumazenil, respectively, conditions for an adequate Stagnara test can be generated if needed. While not exact, raw EEG interpretation by an experienced neurologist is invaluable in determining the depth of anesthesia. The neuromonitoring for this case consisted of a neuromonitoring technician present intraoperatively to follow a raw 4-channel EEG, as well as a neurologist remotely monitoring the case to comment on the depth of anesthesia. Alpha and delta waves are dominant on EEG under typical anesthesia, with increasing delta and theta waveforms emerging with deeper anesthesia [11]. Titrating our infusions down according to the EEG interpretation communicated by the neuromonitoring specialist allowed us to maintain an ideal depth of anesthesia and prevent an obtunding effect from ketamine and midazolam. While ketamine can enhance EEG waveforms, we saw no significant changes in waveforms during the case that were concerning for inadequate anesthetic depth as read by the neuromonitoring team. Finally, we started inhaled anesthetics just before closure for a more predictable wakeup. Volatile anesthetics could not be used for the majority of the case due to its inhibitory effect on the anterior horn cells of the spinal cord that preclude neuromonitoring. Despite previous conflicting recommendations due to concerns for rhabdomyolysis with volatile anesthetic use in VLCADD patients [12], recent literature reports and the review by Redshaw and Stewart have demonstrated that inhaled anesthetics are safe to use in VLCADD patients and were safely used at the end of our case as well [4, 5, 13, 14]. The few existing reports on managing patients with VLCADD recommend strict intraoperative monitoring of glucose and serum CK levels along with continuous glucose infusions to prevent hypoglycemia [12, 14]. Utilizing this strategy, glucose levels ranged from 140 to 200 mg/dL intraoperatively with no significant elevations of CK levels beyond what is expected after a major spine surgery [15]. Had signs of rhabdomyolysis been evident, we would have decreased surgical stress by increasing our anesthetics, transfusing blood products or increasing energy supply through glucose administration.

Finally, pain management, body temperature control, and blood loss were other important considerations. Because pain activates a sympathetic response in the body, it was necessary to provide adequate analgesia with a multimodal approach without obtunding the patient's sensory responses needed for neuromonitoring [4]. Hypothermia was avoided since involuntary muscle movements generate heat and increase skeletal muscle energy demand [5]. Furthermore, the patient's prone positioning during the case and the nature of higher intraoperative blood loss in scoliosis cases required consideration of the risks and benefits of permissive hypotension to decrease blood loss. Given the patient's young age and prior functional status, the risk of excessive bleeding was agreed to be more detrimental than permissive hypotension. Periods of systolic blood pressure below 90 mmHg were met with the assistance of a nicardipine infusion, and tranexamic acid (TXA) was also used to help prevent excessive bleeding. It has been shown that high doses of TXA are safe in the adolescent age group [16]. We bolused 50 mg/kg over 30 minutes, followed by a 25 mg/kg/hr infusion. This aided in our fluid management as well by helping limit blood loss.

Overall, this case demonstrates a distinct and carefully devised anesthetic plan for a patient with unique medical and surgical considerations. The strengths of this case include the sequential use of inhaled anesthetics after TIVA to allow for a rapid wakeup and immediate postoperative physical exam. Additionally, while there have been several reported cases of VLCADD management perioperatively (summarized in Table 2), to our knowledge, intraoperative neuromonitoring in the setting of VLCADD has not been reported. Limitations to our approach include the rarity of VLCADD and the nondefinitive nature of EEG monitoring in signaling the depth of anesthesia to fine-tune our infusions. With communication among anesthesia, surgery, and neuromonitoring teams before and during the operation, the patient successfully underwent a major surgery without complications.

## Figures and Tables

**Table 1:** Intraoperative glucose, CK, lactate, and body temperature measurements.

	Before induction	Start of surgery	Middle of surgery	End of surgery
Glucose (mg/dL)	140	160	176	200
Creatine kinase (U/L)	110	146	322	493
Lactate (mM)	—	1.6	1.5	2.2
Body temperature (C°)	36.8	36.6	37.1	37.8

**Table 2:** Review of literature on VLCADD cases, sorted by year.

Author, year	Number of cases	Patient age (s)	Type of surgery	Anesthetic technique	Adverse events
Bo et al. [17], 2021	1	1.5	Orchiopexy	General anesthesia: induction with fentanyl, rocuronium, and thiopental. Maintenance with sevoflurane	None
Yuasa et al. [5], 2020	1	37	Laparoscopic ovarian cystectomy	General anesthesia: induction with midazolam, remifentanil, and thiamylal. Maintenance with desflurane, remifentanil	None
Hess et al. [13], 2018	1	3	Adenotonsillectomy	General anesthesia with laryngeal mask airway: induction and maintenance with sevoflurane	None
Welsink-Karssies et al. [14], 2016	1	24	Neck cyst removal	General anesthesia: induction with remifentanil, rocuronium, and thiopental. Maintenance with remifentanil and sevoflurane	None
Iwata et al. [18], 2012	1	28	Hysteroscopic myomectomy	General anesthesia: induction with remifentanil, rocuronium, and thiopental. Maintenance with remifentanil and sevoflurane	Slight elevation of lactate intraoperatively, elevated CK on postoperative day 2
Vellekoop et al. [12], 2011	2	8 months, 11 years	Percutaneous endoscopic gastrostomy, placement of intravenous access device (PAC)	Case 1: general anesthesia: induction and maintenance with sevoflurane. Case 2: general anesthesia: induction with fentanyl and propofol. Maintenance with sevoflurane	Case 1: rhabdomyolysis with maximum CK of 163,610U/L and myoglobinuria. Case 2: none
Schmidt et al. [19], 2009	1	8 months	Diagnostic muscle biopsy and percutaneous endoscopic gastrostomy placement	Total intravenous anesthesia: induction with mivacurium, remifentanil, and thiopentone. Maintenance with midazolam and remifentanil	None
Steiner et al. [20], 2002	1	9	Circumcision	General anesthesia with laryngeal mask airway: induction with midazolam and thiopental. Maintenance with alfentanil and midazolam	Delayed awakening (3 hours postoperatively)

## Data Availability

The protected health information used to support the findings of this study are restricted by the MetroHealth System in order to protect patient privacy. Data are available from Dr. Samuel DeJoy MD, sjd5@case.edu, for researchers who meet the criteria for access to confidential data.
